# Systemic Hypertension as a Risk Factor for Open-Angle Glaucoma: A Meta-Analysis of Population-Based Studies

**DOI:** 10.1371/journal.pone.0108226

**Published:** 2014-09-25

**Authors:** Hyoung Won Bae, Naeun Lee, Hye Sun Lee, Samin Hong, Gong Je Seong, Chan Yun Kim

**Affiliations:** 1 Department of Ophthalmology, Severance Hospital, Institute of Vision Research, Yonsei University College of Medicine, Seoul, Korea; 2 Department of Research Affairs, Biostatistics Collaboration Unit, Yonsei University College of Medicine, Seoul, Korea; 3 Department of Ophthalmology, Hallym Hospital, Incheon, Korea; Duke University, United States of America

## Abstract

**Background/Aims:**

Systemic hypertension is thought to increase the risk for developing open-angle glaucoma (OAG) through several mechanisms. However, previous epidemiological studies have shown conflicting results regarding this potential association. We systematically evaluated this issue by conducting a meta-analysis of population-based studies.

**Methods:**

A comprehensive search for articles published before 31 March 2014 was performed using PubMed, Embase, and reference lists. The pooled odds ratio (OR) was calculated using the fixed- and random-effects models, and meta-regression was performed according to age. Subgroup analyses were also conducted, and publication bias was assessed using a funnel plot and Egger’s regression test.

**Results:**

This meta-analysis included 16 studies involving 60,084 individuals, with substantial homogeneity among the studies. The pooled OR for OAG was 1.22 (95% confidence interval, CI: 1.09–1.36) using the fixed-effects model and 1.22 (95% CI: 1.08–1.37) using the random-effects model in all included studies. For subgroup analyses, the pooled OR for high-tension glaucoma (HTG) was higher than that for normal-tension glaucoma (NTG) (OR = 1.92 and 0.94, respectively). No significant difference was detected between Asian and Western populations, and no publication bias was detected in either analysis.

**Conclusions:**

Systemic hypertension increases the risk for developing OAG, especially in those with HTG.

## Introduction

Systemic hypertension (hereafter, hypertension) is a major health issue affecting more than 25% of the adult population worldwide; its prevalence is predicted to affect more than 1.5 billion individuals by 2025 [1], [2]. Hypertension adversely affects not only the heart and kidneys, but is also associated with a wide range of major eye diseases, including glaucoma. Glaucoma is a progressive and irreversible optic neuropathy characterized by visual field loss, and is the second leading cause of blindness worldwide after cataracts [3]. Hypertension is thought to increase the risk of the development and progression of glaucoma [4]–[6], and several mechanisms have been suggested to explain this relationship. Direct microvascular damage caused by hypertension could worsen blood flow to the anterior optic nerve [7]. Autoregulation of the posterior ciliary circulation could also be impaired by hypertension [8]. In addition, antihypertensive therapy could induce hypotensive episodes, especially at night, which could injure the optic nerve [9].

Previous studies have shown incongruous results regarding an association between hypertension and open-angle glaucoma (OAG) [10]–[14]. In the Blue Mountains Eye Study [11], hypertension increased the risk of OAG by more than 50% after adjustment for other glaucoma risk factors such as intraocular pressure (IOP). In addition, the Egna-Neumarkt Study [12] found an association between diagnosis of OAG and hypertension. However, Le et al. [13] found no association between hypertension and OAG; in fact, high blood pressure (BP) showed a trend toward a negative relationship with OAG risk in the Barbados Eye Studies [14]. Therefore, a systematic summary of the relationship between hypertension and OAG is needed.

Most studies that have investigated the association between hypertension and OAG have been observational studies. To the best of our knowledge, no quantitative summary of this association has been published. The overall aim of the present study was to systematically evaluate the association between hypertension and OAG by conducting a meta-analysis of all available and relevant published studies.

## Materials and Methods

### Ethics Statement

This study followed the tenets of the Declaration of Helsinki. The Institutional Review Board of our institute (Yonsei University Health System) determined that this study was exempt from requiring their approval.

### Search Strategy

Two authors (HWB and NL) independently performed systematic PubMed and Embase literature searches of reports published before 31 March 2014. The Preferred Reporting Items of the Systematic Reviews and Meta-Analyses (PRISMA) statement was followed in conducting this meta-analysis [15]. The following search terms were used in PubMed: (“hypertension”[MeSH Terms] OR “hypertension”[All Fields]) AND (“glaucoma”[MeSH Terms] OR “glaucoma”[All Fields]). For Embase, “hypertension”/exp AND “glaucoma”/exp were used as EMTREE terms. All retrieved studies from both PubMed and Embase were exported into CSV files, and overlapping articles were removed manually. The remaining studies were scanned based on titles and abstracts to exclude those that were clearly irrelevant. All inconsistencies were resolved by discussion and review of the original articles. The full texts of the remaining studies were read to check their eligibility. In addition, the reference lists of all identified articles were examined.

### Definition of OAG

OAG was defined as the presence of glaucomatous optic disc change and/or visual field defects regardless of the IOP. Patients with OAG had an open and normal appearing anterior chamber and had no secondary causes. High-tension glaucoma (HTG) was defined in patients with OAG with an IOP≥22 mmHg, and normal-tension glaucoma (NTG) was defined in patients with OAG with an IOP<22 mmHg.

### Inclusion and Exclusion Criteria

Studies included in this meta-analysis (1) were population-based, (2) evaluated hypertension and OAG, and (3) reported odds ratios (ORs) with 95% confidence intervals (CIs) to measure the association between hypertension and OAG or showed the raw data in the article to calculate the OR with 95% CI. Studies excluded from this meta-analysis (1) were not reported in English, (2) did not include hypertension as a risk factor for OAG, (3) covered angle-closure glaucoma or secondary glaucoma or did not clearly designate OAG, or (4) defined hypertension or glaucoma based only medical records, questionnaire, or self-reported history. When multiple publications reported the same population, only the most recent study was included.

### Data Extraction and Quality Assessment

Data were extracted from the studies by two independent authors (HWB and NL). Disagreement was settled by discussion and review of the articles. The following information was extracted for each study: (1) the first author’s last name, (2) year of publication, (3) country of study, (4) name of study population (if available), (5) study design, (6) number of subjects (7) mean age of subjects, (8) definition of hypertension, (9) type of glaucoma, (10) effect size (OR and 95% CI), and (11) confounders used for adjustment. We calculated the OR from raw data when no OR was provided in the report. In most reports with more than one effect size, we chose the OR adjusted for the largest number of confounding factors. However, two ORs were extracted from the Andhra Pradesh Eye Disease Study, in which urban and rural cohorts were completely separated [16]. The study quality was assessed by inverse-variance weighting.

### Statistical Analysis

The pooled ORs with 95% CIs were estimated using the fixed- and the random-effects models. The fixed-effects model is useful when analyzing a sufficiently homogeneous group of studies, whereas the random-effects model is more appropriate when there is heterogeneity among individual studies [17]. Heterogeneity among studies was identified visually using a forest plot and quantified statistically using the I^2^ index. The I^2^ index can be interpreted as the percentage of the total variability in a set of effect sizes due to true heterogeneity between studies [18]. Sensitivity analysis was also performed to evaluate the stability of the meta-analysis. Omitting one study at a time, the pooled OR for the remaining studies was calculated and compared to that of the fixed- or random-effects model. Publication bias was evaluated using Egger’s regression test and/or a funnel plot [19]. In addition, meta-regression analysis was performed using age, which could affect the prevalence of both hypertension and OAG. Subgroup meta-analysis was carried out to evaluate each pooled OR in more homogeneous subgroups and to identify characteristics of the association between hypertension and OAG.

All statistical analyses were performed using Comprehensive Meta-Analysis version 2.0 (Biostat, Inc., Englewood, NJ). A two-sided *P* value of <0.05 was considered to indicate statistical significance.

## Results

**Figure 1:**
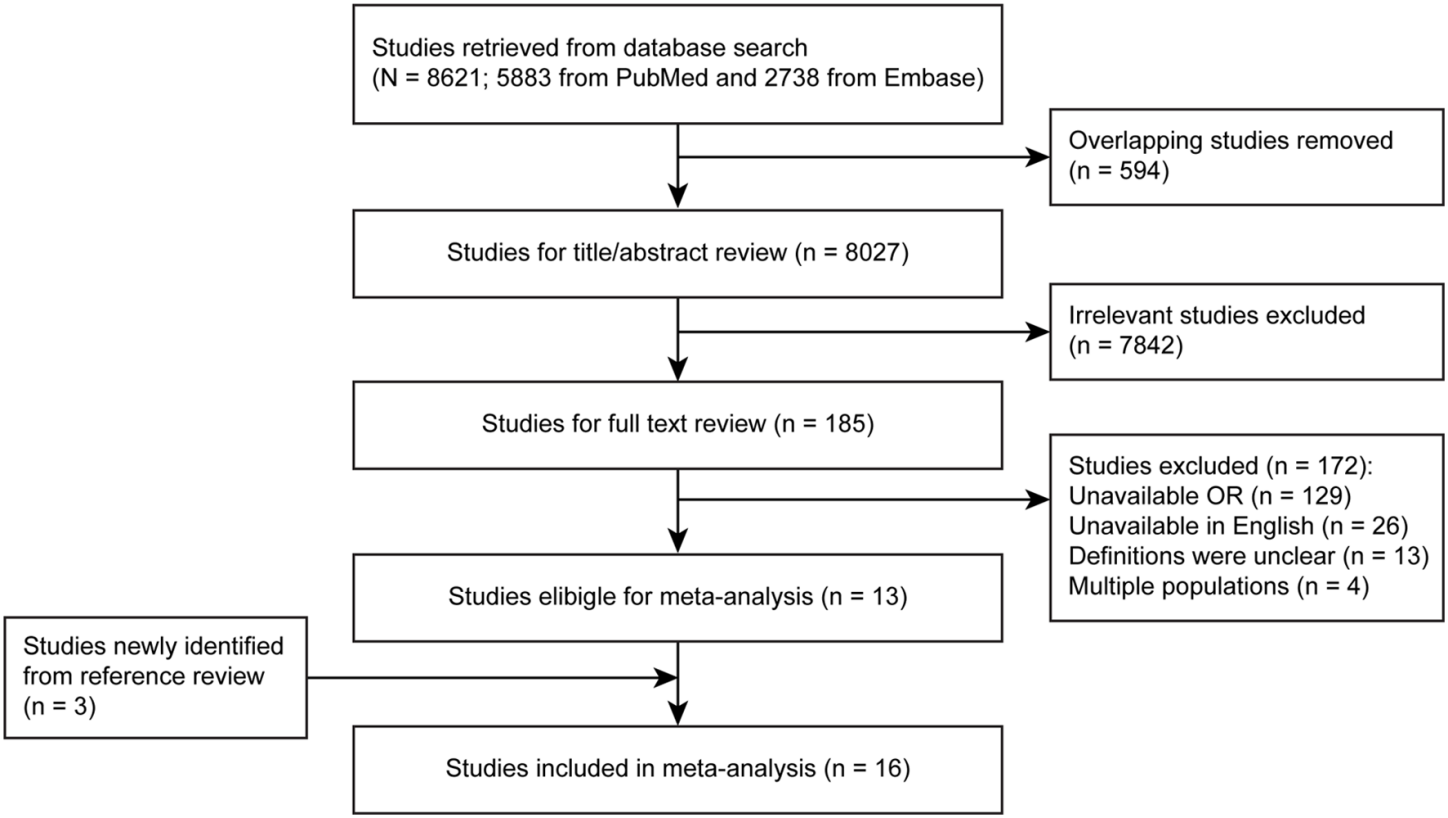
Flow chart of the literature search and selection criteria for inclusion in the meta-analysis. OR, odds ratio; OAG, open-angle glaucoma.

The primary literature search in PubMed and Embase identified 8621 studies. After overlapping and irrelevant studies were excluded through a title and abstract review, 185 potentially relevant studies were investigated using a full-text review. Of these studies, 13 met the inclusion criteria for the meta-analysis, and 3 additional eligible studies were found from the reference review. Finally, 16 studies were enrolled in this meta-analysis (**Figure 1**) [11], [12], [14], [16], [20]–[31].

**Table 1** shows the characteristics of the enrolled studies. A total of 60,084 subjects were included in this meta-analysis. The mean ages of the included populations ranged from 51.0 to 70.8 years. The studies were published between 1995 and 2012, and all studies were population-based: 15 were cross-sectional studies [11], [12], [16], [20]–[31] and one was a longitudinal cohort study [14]. Nine studies were performed on Asian populations [16], [22]–[24], [26]–[29], [31] and seven investigated Western populations [11], [12], [14], [20], [21], [25], [30]. Hypertension was defined based on the use of antihypertensive medication and/or the actual BP in all studies. Two studies [12], [25] indicated the ORs for OAG, HTG, and NTG, while the remaining studies presented the OR for OAG only.

**Table 1:** Characteristics of the Population-based Studies Included in the Meta-analysis.

First author, Year	Country(study name)	Studydesign	No. ofsubjects	Meanage (years)	Definitionof hypertension	Type ofglaucoma*	OR(95% CI)	Adjustedcovariates
Tielsch, 1995 [20]	USA (The Baltimore Eye Survey)	Cross-sectional	5305	60.6	Medication or SBP of ≥160 mmHg and/or DBP of ≥95 mmHg	OAG	0.99 (0.72–1.37)	Age, race
Bonomi, 2000 [12]	Italy (The Egna-Neumarkt Study)	Cross-sectional	4171	59.1	Medication or SBP of ≥160 mmHg and/or DBP of ≥95 mmHg	OAG	1.4 (0.9–2.3)	Age, sex
			4147	59.0		HTG	2.1 (1.2–3.6)	
			4111	59.0		NTG	0.6 (0.2–1.4)	
Quigley, 2001 [21]	USA (Project VER)	Cross-sectional	4764	57.1	Medication or SBP of ≥160 mmHg and/or DBP of ≥90 mmHg	OAG (the ISGEO classification)	0.97 (0.63–1.48)	Age
Ramakrishnan, 2003 [22]	India (The Aravind Comprehensive Eye Survey)	Cross-sectional	5150	51.0	Medication or SBP of ≥160 mmHg and/or DBP of ≥90 mmHg	OAG	1.00 (0.50–2.00)	Age, sex, DM, pseudoexfoliation, myopia
Mitchell, 2004 [11]	Australia (The Blue Mountain Eye Study)	Cross-sectional	3627	66.2	Medication or SBP of ≥160 mmHg and/or DBP of ≥95 mmHg	OAG	1.56 (1.01–2.40)	age, sex, IOP, family history, myopia, DM, pseudoexfoliation, thyroxine use
Vijaya, 2005 [23]	India (The Chennai Glaucoma Study; Rural)	Cross-sectional	3924	53.8	Medication or SBP of ≥140 mmHg and/or DBP of ≥90 mmHg	OAG (the ISGEO classification)	1.02 (0.60–1.74)	Age, sex, IOP, CCT, myopia
Suzuki, 2006 [24]	Japan (The Tajimi Study)	Cross sectional	2852	58.0	Medication or SBP of ≥160 mmHg and/or DBP of ≥95 mmHg	OAG	1.67† (1.14–2.43)	
Hulsman, 2007 [25]	Netherlands (The Rotterdam Study)	Cross-sectional	5317	69.0	Medication or SBP of ≥160 mmHg and/or DBP of ≥100 mmHg	OAG	1.29 (0.92–1.81)	Age, sex, DM, cholesterol, BMI, smoking
			5167	68.9		HTG	1.72 (0.95–3.12)	
						NTG	1.10 (0.73–1.66)	
			5252	68.9				
Leske, 2008 [14]	Barbados (The Barbados Eye Study)	Cohort study	3214	56.9	Medication or SBP of ≥140 mmHg and/or DBP of ≥90 mmHg	OAG	1.36† (0.97–1.92)	
Vijaya, 2008 [26]	India (The Chennai Glaucoma Study; Urban)	Cross-sectional	3850	54.8	Medication or SBP of ≥140 mmHg and/or DBP of ≥90 mmHg	OAG (the ISGEO classification)	1.1 (0.7–1.6)	Age, sex, IOP, CCT, myopia
Wang, 2009 [27]	China (The Beijing Eye Study)	Cross-sectional	3221	60.4	Medication or SBP of ≥140 mmHg and/or DBP of ≥90 mmHg	OAG	1.35 (0.86–2.14)	
Tan, 2009 [28]	Singapore (The Singapore Malay Eye Study)	Cross-sectional	3279	58.7	Medication or SBP of ≥135 mmHg and/or DBP of ≥85 mmHg	OAG (the ISGEO classification)	0.72 (0.45–1.17)	Age, sex, education, smoking, CCT, DM treatment
Garudadri, 2010 [16]	India (The Andhra Pradesh Eye Disease Study)	Cross-sectional	934 (Urban)	53.2	Medication or SBP of ≥140 mmHg and/or DBP of ≥90 mmHg	OAG (the ISGEO classification)	0.97 (0.45–2.06)	Age, sex, IOP, myopia, DM
			2970 (Rural)	54.7			1.20 (0.65–2.20)	
Ishikawa, 2011 [29]	Japan	Cross-sectional	710	54.7	SBP of ≥140 mmHg or DBP of ≥90 mmHg	OAG	1.07 (0.41–2.78)	age, sex, DBP, IOP, OPP
Topouzis, 2011 [30]	Greece (The Thessaloniki Eye Study)	Cross-sectional	1840	70.8	SBP of ≥140 mmHg or DBP of ≥90 mmHg	OAG	1.43 (0.70–2.91)	Age, IOP, DM, coronary artery surgery, myopia
Sun, 2012 [31]	China	Cross-sectional	4956	57.0	Medication or SBP of ≥140 mmHg and/or DBP of ≥90 mmHg	OAG (the ISGEO classification)	2.45 (1.17–5.16)	Age, family history, IOP

*OAG = HTG and NTG; HTG = IOP≥22 mmHg; NTG = IOP<22 mmHg

† OR calculated from the raw data in the article.

‡ Matched variables between cases and controls.

OR, odds ratio; CI, confidence interval; N/A, not applicable; OAG, open-angle glaucoma; HTG, high tension glaucoma; NTG, normal-tension glaucoma; IOP, intraocular pressure; SBP, systolic blood pressure; DBP, diastolic blood pressure; DM, diabetes mellitus; ISGEO, International Society of Geographical and Epidemiological Ophthalmology [39]; CCT, central corneal thickness; BMI, body mass index; OPP, ocular perfusion pressure.

**Figure 2:**
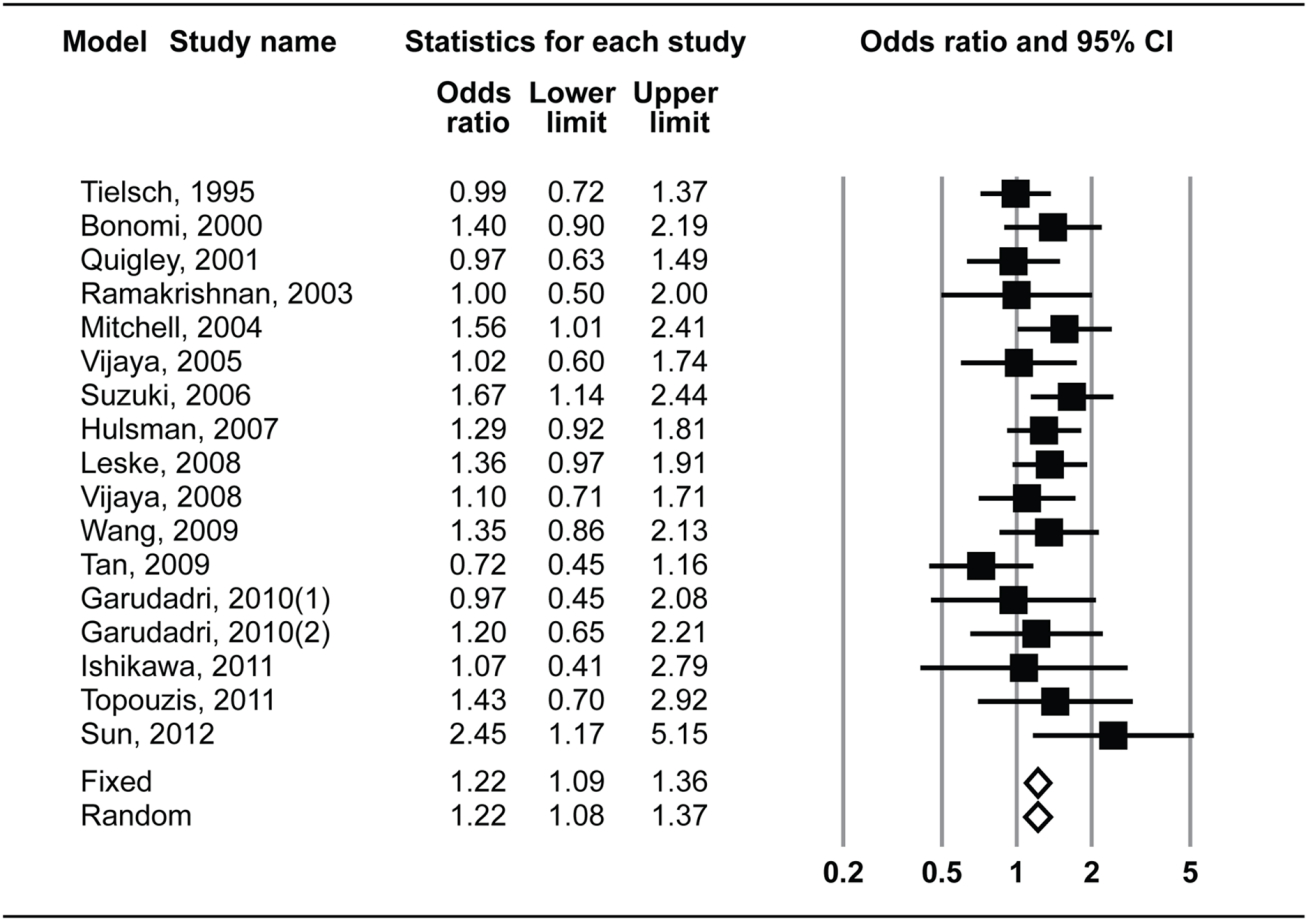
Forest plot of risk estimates for the association between systemic hypertension and open-angle glaucoma in all included studies. CI, confidence interval.

**Figure 3:**
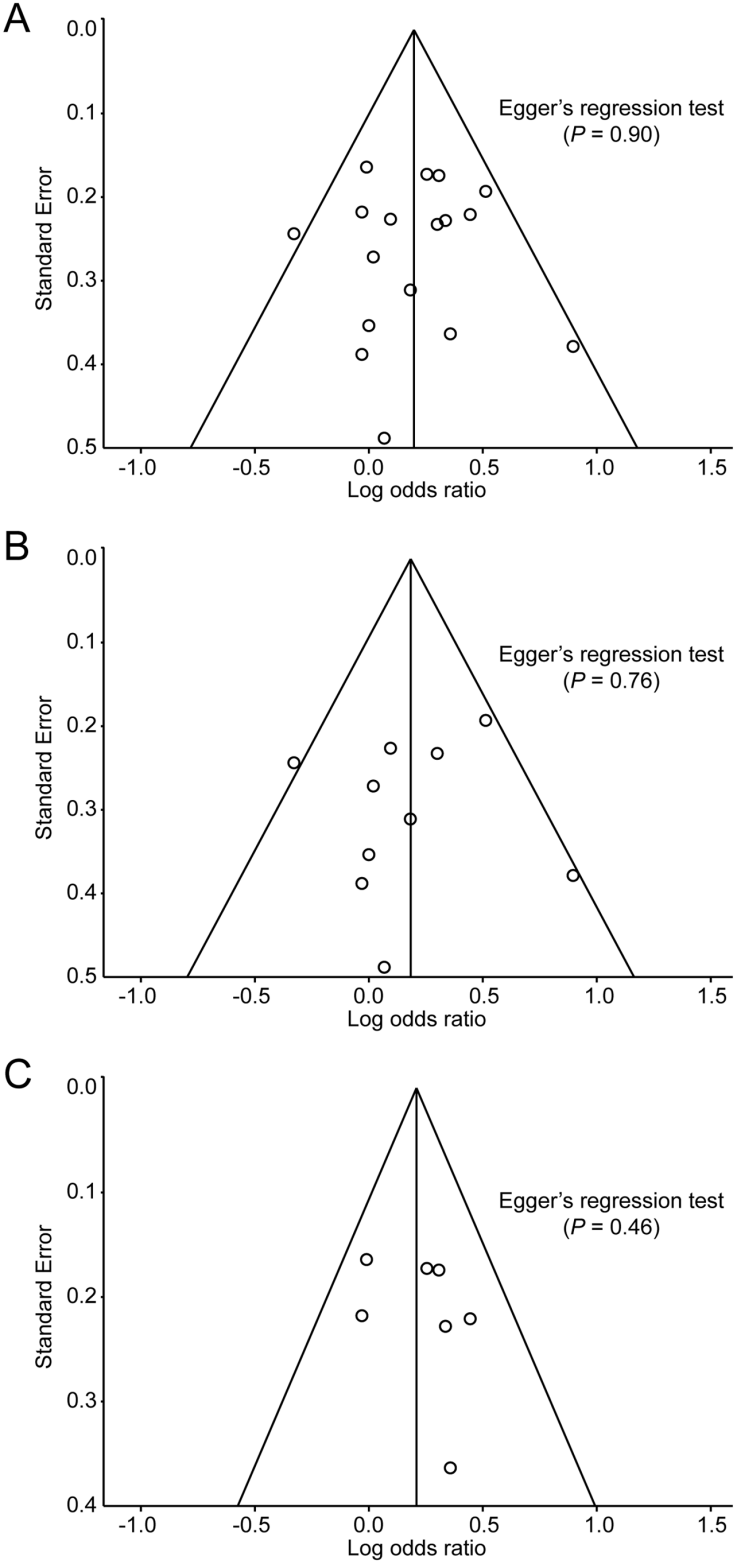
Funnel plots of the odds ratio of developing open-angle glaucoma for identifying publication bias. Odds ratios are displayed on a logarithmic scale. A, all included populations; B, Asian populations; C, Western populations.

**Figure 4:**
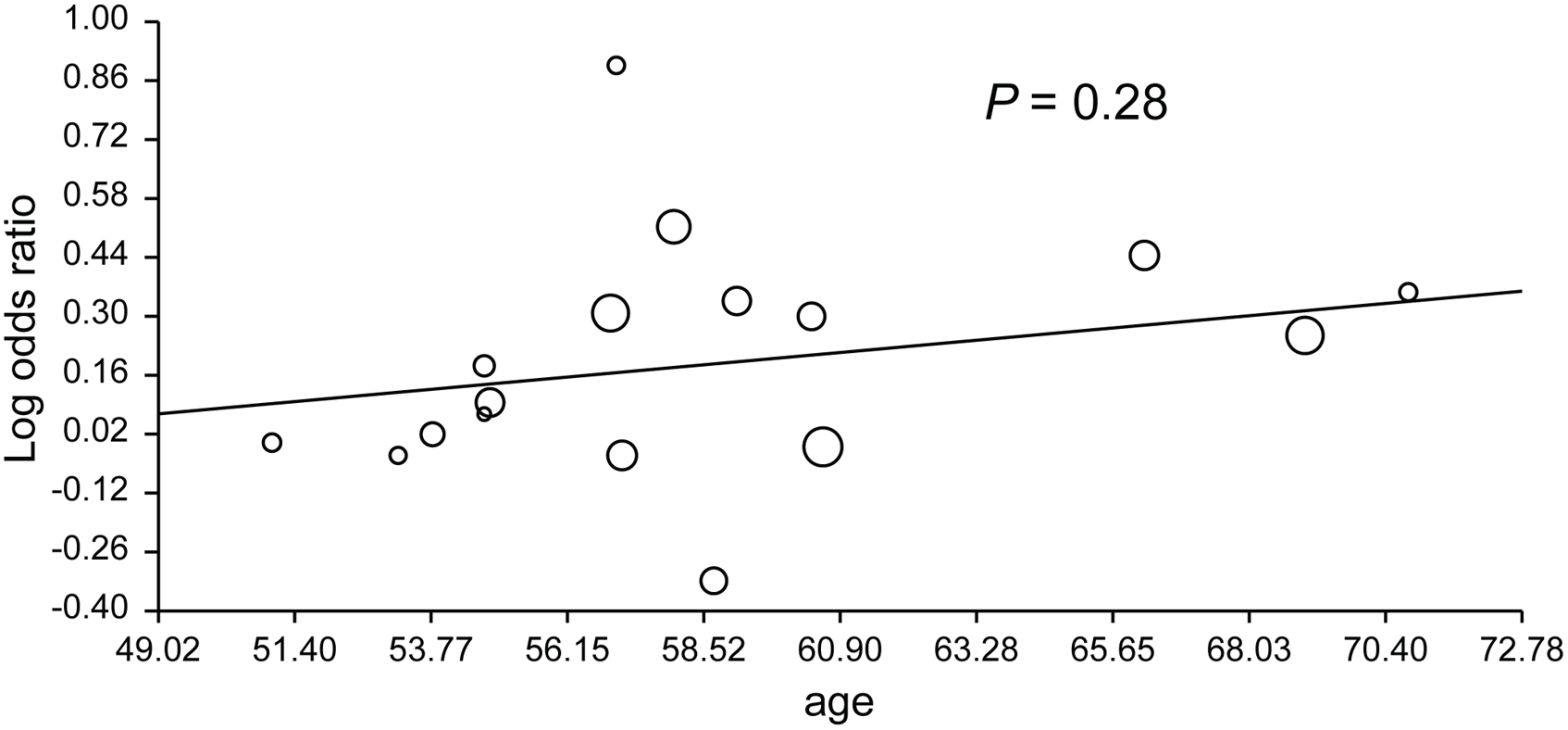
Meta-regression analysis between the pooled odds ratio and age for open-angle glaucoma in all included studies. Odds ratios are displayed on a logarithmic scale (*P* = 0.28).

The forest plot and I^2^ index indicated statistically significant homogeneity (I^2^ = 7.5%, *P* = 0.37) among all included studies for OAG. The pooled OR was 1.22 (95% CI: 1.09–1.36) using the fixed-effects model and 1.22 (95% CI: 1.08–1.37) using the random-effects model (**Figure 2**). Sensitivity analysis showed that no study had a strong influence on the pooled OR in this meta-analysis (**Table S1**), and the funnel plot and Egger’s regression test (*P* = 0.90) indicated no obvious evidence of publication bias (**Figure 3A**). Meta-regression analysis showed no significant association between age and pooled OR (*P* = 0.28) (**Figure 4**).

**Figure 5:**
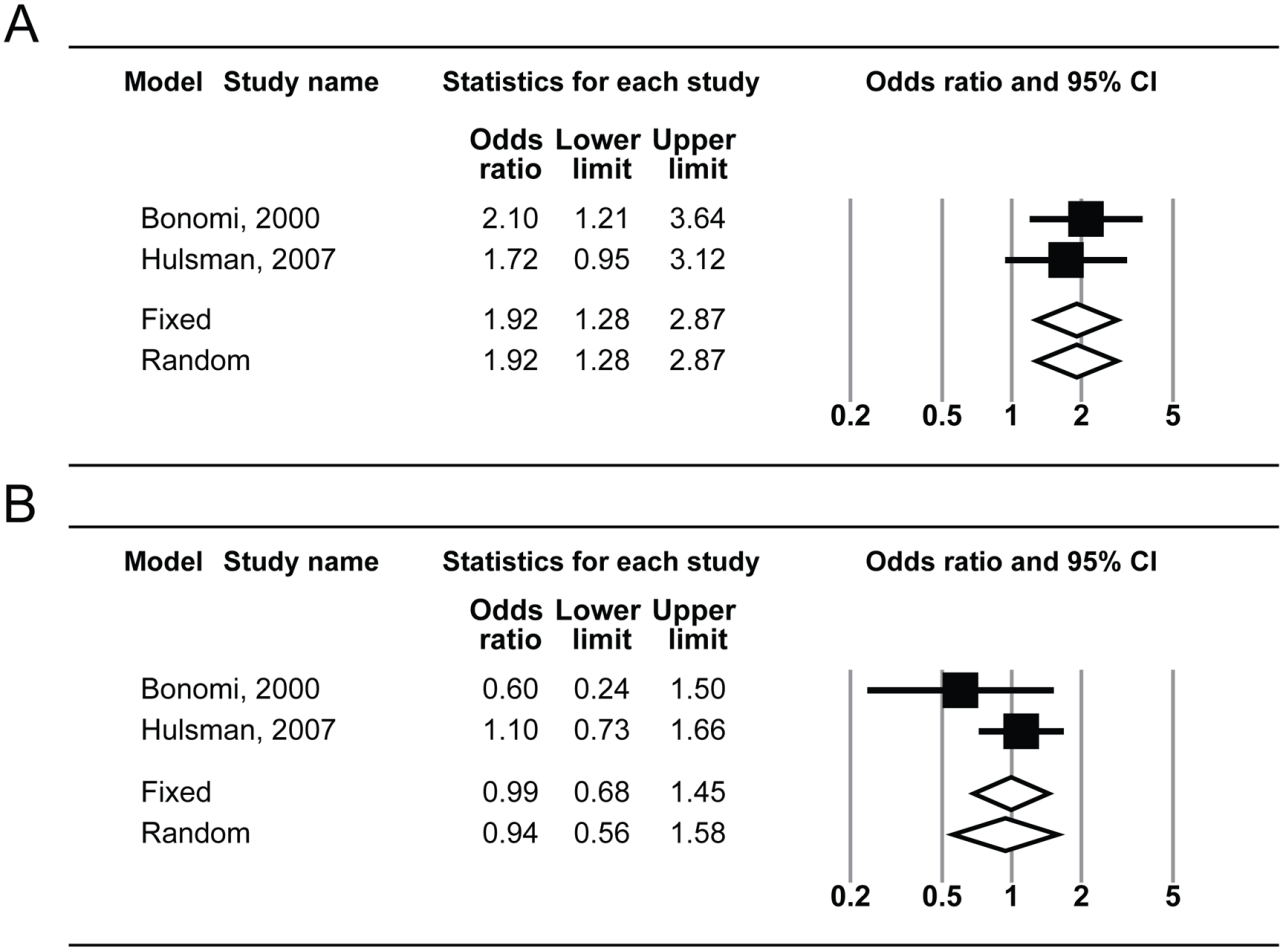
Subgroup analysis showing forest plot of risk estimates for the association between systemic hypertension and high-tension glaucoma (top, A) and systemic hypertension and normal-tension glaucoma (bottom, B). CI, confidence interval.

**Figure 6:**
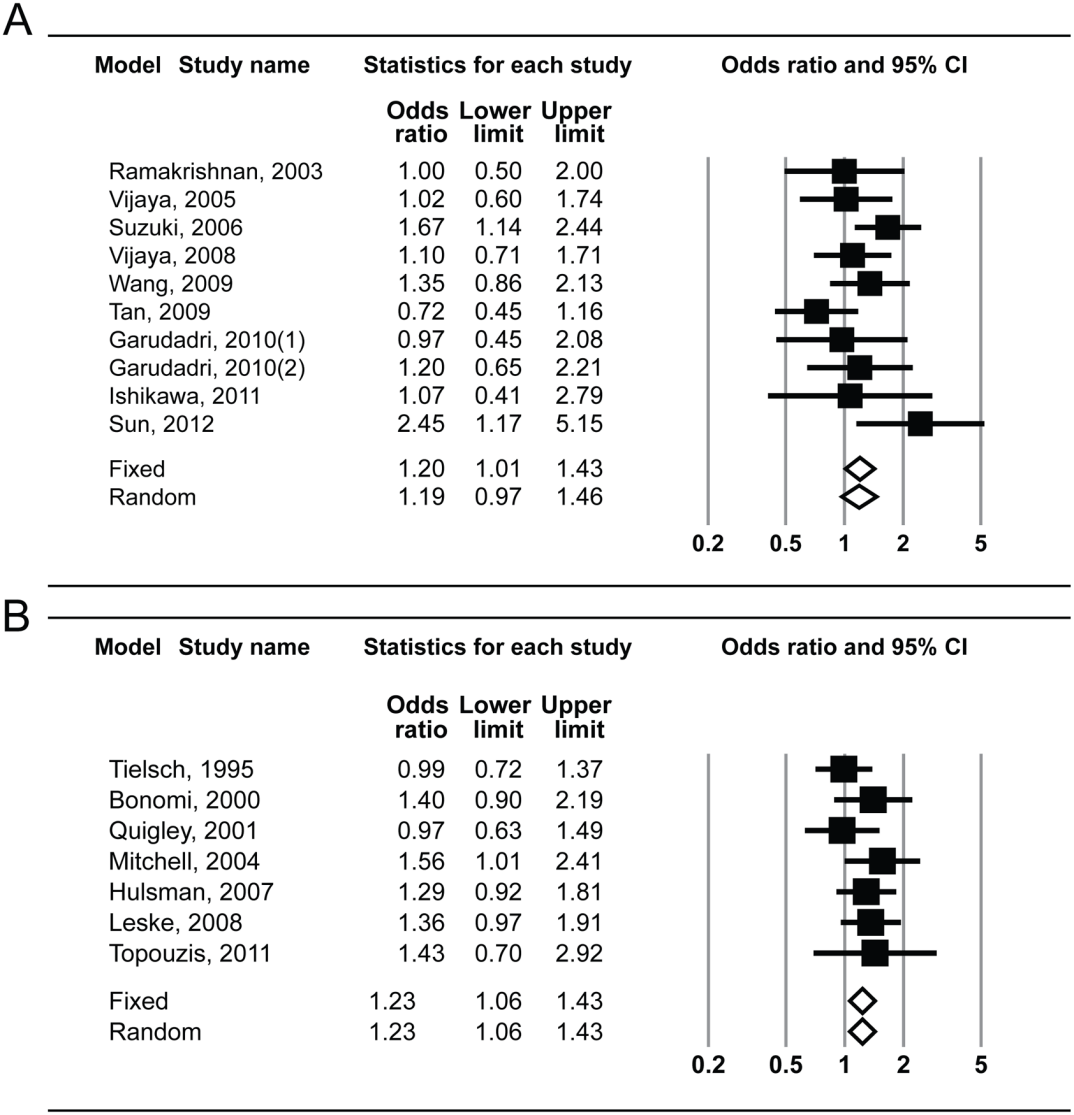
Subgroup analysis showing forest plot of risk estimates for the association between systemic hypertension and open-angle glaucoma in Asian (top, A) and Western populations (bottom, B). CI, confidence interval.

For the subgroup analysis, the pooled OR for HTG (heterogeneity; I^2^ = 0.0%) was 1.92 (95% CI: 1.28–2.87) using the fixed-effects model, whereas that of NTG (heterogeneity; I^2^ = 28.4%) was 0.99 (95% CI: 0.68–1.45) using the fixed-effects model and 0.94 (95% CI: 0.56–1.58) using the random-effects model (**Figure 5**). The pooled OR from Asian populations (heterogeneity; I^2^ = 26.6%) was 1.20 (95% CI: 1.01–1.43) using the fixed-effects model and 1.19 (95% CI: 0.97–1.46) using the random-effects model, whereas that from Western populations (heterogeneity; I^2^ = 0.0%) was 1.23 (95% CI: 1.06–1.43) using the fixed-effects model (**Figure 6**). No evidence of publication bias was detected by Egger’s regression test (*P* = 0.76 and 0.46, respectively) or the funnel plot (**Figure 3B and 3C**).

## Discussion

The present meta-analysis of population-based studies showed that individuals with hypertension have an approximately 1.2-fold higher risk of developing OAG than individuals without hypertension. The pooled OR was 1.22 (95% CI: 1.09–1.36) using the fixed-effects model and 1.22 (95% CI: 1.08–1.37) using the random-effects model.

To maintain homogeneity among the included studies and to minimize the possibility of selection bias, we enrolled only population-based studies in this meta-analysis. Hospital-based studies (which were mostly case-control studies) showed selection bias, potentially because the individuals with hypertension had a higher chance of greater access to the medical care system. Thus, our meta-analysis maintained a high degree of homogeneity among the studies (I^2^ = 7.5%, *P* = 0.37).

Age is one of the most important confounding factors affecting the prevalence of hypertension and OAG. The meta-regression model was used to examine the impact of age on the effect size. The results showed that age was not significantly associated with the ORs in this meta-analysis (*P* = 0.28). Therefore, hypertension increased the risk of OAG development irrespective of age.

OAG has been considered a single disease because of the similar properties between HTG and NTG [32]–[34]. In this study, we performed subgroup meta-analyses for HTG and NTG to evaluate the effects of IOP. Although only two studies were enrolled, we found that the risk of developing HTG was increased to a greater extent by hypertension than that of OAG, whereas the risk of developing NTG was not affected by hypertension (OR = 1.92 and 0.94, respectively). This difference might be attributable to the difference in IOP, which is related to ocular perfusion pressure (OPP). Hypertension could be associated with the development of OAG via two mechanisms. One mechanism could be that increased BP leads to reduced ocular blood flow due to thickening and stiffening of the vessels wall, therefore increasing the risk of OAG [35]. The second mechanism is that a higher BP produces a higher OPP, which could decrease the risk of OAG [36]. Because OPP can be calculated as 2/3[(systolic BP+2diastolic BP)/3] – IOP, it can be increased by high BP or low IOP. If two subgroups had similarly high BPs, individuals with HTG might have lower OPP than those with NTG. Therefore, it is thought that the increased risk induced by hypertension might be compensated by high OPP in individuals with NTG, and might be strengthened by low OPP in those with HTG.

Another subgroup analysis according to country showed no difference between Asian and Western populations. The ORs of Asian and Western populations were both about 1.2, similar to that for the total included population. Because NTG is more common in Eastern than Western populations [37], it was expected that the risk of developing OAG would be higher in the Western than in the Asian population. However, this meta-analysis showed no significant regional differences, although the OR in Western populations was slightly higher than that in Asian populations. Further studies considering BP and IOP are required to evaluate the regional or ethnic differences regarding the relationship between hypertension and OAG.

This meta-analysis had several limitations originating from the individual studies and the meta-analysis itself. First, although the funnel plot and Egger’s regression test demonstrated no evidence of publication bias, our analyses were not entirely free from several types of reporting bias [38]. For example, the possibility of location, citation, and language biases could not be excluded. Second, other unrevealed confounding factors or inadequate control of such factors might have affected the ORs. Although we found that age (the most powerful confounding factor) showed no association with the OR using meta-regression, we did not consider all confounding factors. In addition, adjusted confounding factors in each study varied among the included studies, and some ORs were calculated from the raw data without adjustment. This uneven management of confounding factors could have led to overestimation or underestimation of the ORs. Finally, we could not elucidate the precise correlation between OAG and the actual BP or type of antihypertensive medication. However, this study has clinical significance in that we targeted patients in our clinic who were considered to have hypertension.

To the best of our knowledge, this study is the first meta-analysis of the association between hypertension and OAG. This study addressed the controversial issue regarding hypertension as a risk factor for OAG and revealed that hypertension increases the risk of developing OAG. Moreover, we revealed contradictory results between HTG and NTG through the subgroup analysis. Individuals with hypertension had the highest risk of developing HTG because of low OPP induced by high IOP. Clinicians should be aware of this possibility and give appropriate advice about glaucoma evaluation to patients with hypertension.

In conclusion, the present meta-analysis of population-based studies showed that systemic hypertension is a risk factor for OAG. Although further research is needed, OAG should be taken into account when treating individuals with hypertension.

## Supporting Information

Table S1 Analysis of the sensitivity of all included studies in the random-effects model.
(DOC)

Checklist S1 **PRISMA checklist.**
(DOC)

Diagram S1 **PRISMA flow chart.**
(DOC)
