# Supplementary material for: Systemic Hypertension as a Risk Factor for Open-Angle Glaucoma: A Meta-Analysis of Population-Based Studies
Source: PLoS One. 2014 Sep 25;9(9):e108226. doi: 10.1371/journal.pone.0108226 (PMC4177901; doi:10.1371/journal.pone.0108226)
Supplement: Table S1 — Analysis of the sensitivity of all included studies in the random-effects model. (DOC) [file pone.0108226.s001.doc]

**Table S1.** Analysis of the sensitivity of all included studies in the random-effects model.

| **Study excluded** | **OR (95% CI)** | **Heterogeneity [I^2^ index (%)]** | ***P* value** |
| --- | --- | --- | --- |
| None | 1.22 (1.08–1.37) | 7.5% | 0.37 |
| Tielsch, 1995 [20] | 1.25 (1.11–1.42) | 3.1% | 0.42 |
| Bonomi, 2000 [12] | 1.21 (1.07–1.37) | 11.3% | 0.32 |
| Quigley, 2001 [21] | 1.24 (1.10–1.40) | 7.0% | 0.37 |
| Ramakrishnan, 2003 [22] | 1.23 (1.08–1.39) | 11.7% | 0.32 |
| Mitchell, 2004 [11] | 1.20 (1.06–1.35) | 6.1% | 0.38 |
| Vijaya, 2005 [23] | 1.23 (1.09–1.39) | 11.0% | 0.33 |
| Suzuki, 2006 [24] | 1.18 (1.05–1.33) | 0.0% | 0.50 |
| Hulsman, 2007 [25] | 1.21 (1.06–1.38) | 12.7% | 0.31 |
| Leske, 2008 [14] | 1.20 (1.06–1.37) | 11.1% | 0.33 |
| Vijaya, 2008 [26] | 1.23 (1.08–1.39) | 12.2% | 0.31 |
| Wang, 2009 [27] | 1.21 (1.07–1.37) | 12.3% | 0.31 |
| Tan, 2009 [28] | 1.26 (1.12–1.41) | 0.0% | 0.65 |
| Garudadri, 2010 (Urban) [16] | 1.23 (1.08–1.39) | 11.5% | 0.32 |
| Garudadri, 2010 (Rural) [16] | 1.22 (1.08–1.38) | 13.3% | 0.30 |
| Ishikawa, 2011 [29] | 1.22 (1.08–1.38) | 13.0% | 0.31 |
| Topouzis, 2011 [30] | 1.21 (1.07–1.37) | 12.3% | 0.31 |
| Sun, 2012 [31] | 1.20 (1.07–1.34) | 0.0% | 0.54 |

OR, odds ratio; CI, confidence interval.
