# Supplementary material for: Systemic Hypertension as a Risk Factor for Open-Angle Glaucoma: A Meta-Analysis of Population-Based Studies
Source: PLoS One. 2014 Sep 25;9(9):e108226. doi: 10.1371/journal.pone.0108226 (PMC4177901; doi:10.1371/journal.pone.0108226)
Supplement: Diagram S1 — PRISMA flow chart. (DOC) [file pone.0108226.s003.doc]

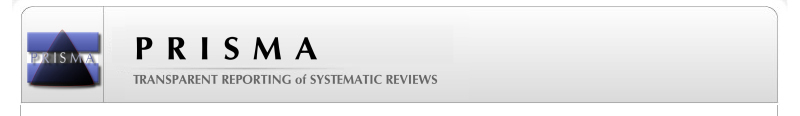
**PRISMA 2009 Flow Diagram**

**Screening**

**Included**

**Eligibility**

**Identification**

Records identified through database searching
(n =7948)

Additional records identified through other sources
(from reference review, n = 3 )

Records after duplicates removed
(n = 564 )

Records screened
(n = 7351 )

Records excluded
(n = 7206 )

Full-text articles assessed for eligibility
(n = 181 )

Full-text articles excluded, with reasons
(n = 156 )

Studies included in qualitative synthesis
(n = 25 )

Studies included in quantitative synthesis (meta-analysis)
(n = 25 )
